# Molecular Mechanisms Underlying the *Claviceps purpurea*–*Secale cereale* Interaction: From Floral Biotrophy to Ergot Alkaloid Biosynthesis

**DOI:** 10.3390/ijms27167369

**Published:** 2026-08-18

**Authors:** Francisca Sempere-Ferre, Celia Almela-Camañas

**Affiliations:** 1 Departamento de Estadística e Investigación Operativa Aplicadas y Calidad, Universitat Politècnica de València, Camino de Vera s/n, 46022 Valencia, Spain; 2 Departamento de Nutrición, Facultad de Medicina y Ciencias de la Salud, Universidad Católica de Valencia San Vicente Mártir, c/Quevedo, 2, 46001 Valencia, Spain

**Keywords:** *Claviceps purpurea*, floral biotrophy, host–pathogen interaction, ergot alkaloids, fungal differentiation, secondary metabolism, multi-omics approaches, plant–fungus interaction

## Abstract

*Claviceps purpurea* is a highly specialized biotrophic ascomycete that colonizes floral tissues of grasses, including economically important cereal crops, causing ergot disease and producing ergot alkaloids with significant agricultural, pharmaceutical, and biotechnological relevance. Despite extensive research on its biology and secondary metabolism, the molecular mechanisms underlying host recognition, floral specificity, establishment of biotrophy, and developmental differentiation remain incompletely understood. This review integrates current knowledge derived from genomic, transcriptomic, proteomic, metabolomic, and functional genetic studies to provide an overview of the molecular basis of the *C. purpurea*–host interaction. Particular emphasis is placed on recent advances in fungal development, host immune modulation, hormonal signalling, sclerotial differentiation, and ergot alkaloid biosynthesis. Current evidence indicates that successful colonization depends on coordinated regulation of host recognition, secretion of effector proteins, carbohydrate-active enzymes, and manipulation of host signalling pathways to establish and maintain a biotrophic lifestyle. The transition from the sphacelial stage to sclerotial development represents a major developmental and metabolic reprogramming event associated with fungal differentiation and activation of the ergot alkaloid biosynthetic pathway. Recent multi-omics approaches have further revealed complex regulatory networks connecting fungal development and secondary metabolism. *Claviceps purpurea* has emerged as a valuable model for studying floral biotrophy and fungal secondary metabolism; however, key questions remain regarding the molecular basis of host specificity, effector function, hormonal crosstalk, and developmental regulation. Future integration of multi-omics approaches with functional genomics will be essential to resolve these processes and to support sustainable disease management strategies and the biotechnological exploitation of ergot alkaloids.

## 1. Introduction

*Claviceps purpurea* (Fr.) Tul. is a biotrophic ascomycete belonging to the family Clavicipitaceae and is the causal agent of ergot disease in numerous cereal crops and wild grasses. The pathogen is widely distributed throughout temperate regions and primarily infects members of the Poaceae, including rye, wheat, barley and several forage grasses. In addition to reducing grain yield and quality, infection results in the replacement of developing grains by dark sclerotia containing ergot alkaloids, a diverse group of bioactive secondary metabolites responsible for ergotism in humans and livestock. Conversely, these compounds also constitute valuable pharmaceutical precursors owing to their diverse biological activities [1,2].

Beyond its agronomic importance, *C. purpurea* has become an important experimental model for investigating fungal development, host specialization, biotrophic plant–fungus interactions and the regulation of secondary metabolism. Unlike many fungal pathogens that colonize vegetative tissues, *C. purpurea* exhibits a remarkable degree of floral specialization, infecting reproductive tissues and establishing a highly adapted interaction with the host ovary that has attracted considerable attention in plant pathology and fungal biology [2,3].

During the last two decades, the availability of genome sequences together with advances in transcriptomics, proteomics and metabolomics has greatly expanded our understanding of the molecular mechanisms underlying host colonization, fungal differentiation and ergot alkaloid biosynthesis. These approaches have identified regulatory networks involved in fungal development and provided new insights into the molecular dialogue established between *C. purpurea* and its cereal hosts. Nevertheless, several fundamental aspects of this interaction remain incompletely understood, including the molecular mechanisms that enable the fungus to establish a compatible biotrophic relationship within the host ovary, manipulate plant immune and hormonal signalling, and coordinate infection with sclerotial differentiation and ergot alkaloid biosynthesis. [3,4].

Although previous reviews have provided valuable insights into ergot disease, *Claviceps* biology, and ergot alkaloid biosynthesis, recent advances in functional genomics and molecular genetics have revealed new regulatory mechanisms controlling host colonization, fungal differentiation, and secondary metabolism. However, these molecular findings have not yet been comprehensively integrated into a framework connecting gene regulation, developmental transitions, and host–fungus interactions throughout the *C. purpurea* infection cycle. This review therefore provides an updated molecular perspective, highlighting functional studies and the regulatory networks underlying floral biotrophy, sclerotial development, and ergot alkaloid production.

**Figure 1:**
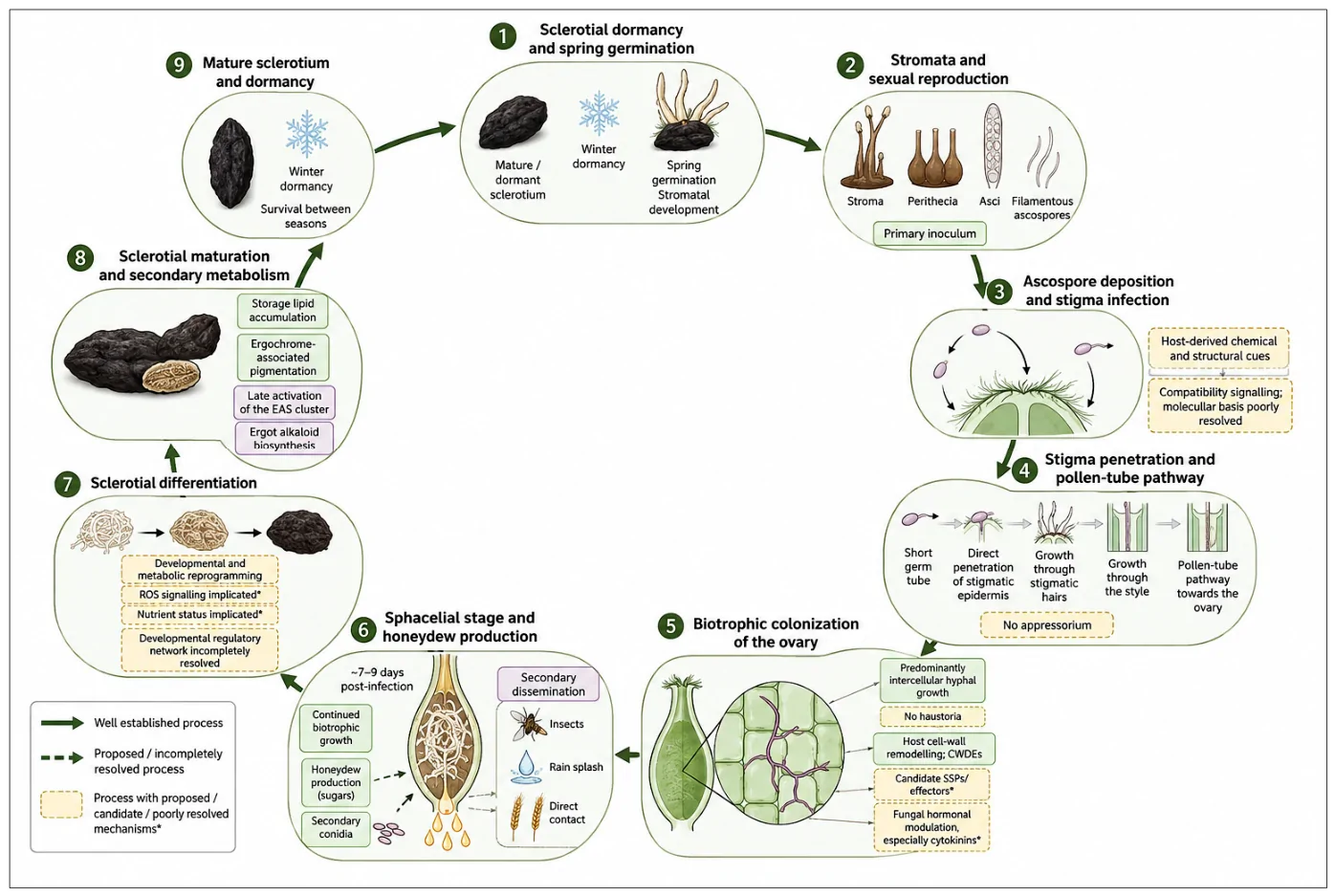
Overview of the life cycle of *Claviceps purpurea* and the major molecular processes involved in host recognition, floral colonization, establishment of biotrophy, honeydew production, sclerotial differentiation, ergot alkaloid biosynthesis, dormancy, and sexual reproduction. The schematic illustration was conceptualized by the authors and generated with the assistance of OpenAI ChatGPT (GPT-5.5/DALL·E), followed by manual editing and scientific validation by the authors. Yellow boxes indicate proposed, candidate, or poorly resolved mechanisms. Green and purple backgrounds are used for visual organization only and do not indicate additional biological categories or levels of evidence. * Proposed, candidate, or poorly resolved mechanisms.

In this context, this manuscript integrates current knowledge on the biology of *Claviceps purpurea*, with particular emphasis on the molecular mechanisms underlying host recognition, biotrophic establishment, fungal development, and secondary metabolism. In addition, we highlight recent advances in genomic, transcriptomic, metabolomic and functional genetic studies and discuss major unresolved questions that are expected to shape future research on this unique plant–fungus interaction. Given the increasing availability of multi-omics datasets and functional genomic resources, an integrated understanding of floral biotrophy and secondary metabolism in *C. purpurea* is both timely and necessary. Although many of the examples discussed in this review are derived from the *C. purpurea*–rye interaction, the molecular mechanisms described are broadly relevant to infections of other susceptible cereal crops and grasses. The manuscript is organized into five main sections that address the fungal life cycle, the molecular mechanisms underlying host colonization and immune modulation, the transition from biotrophic growth to sclerotial differentiation, the regulation of ergot alkaloid biosynthesis, and future perspectives for understanding this highly specialized plant–fungus interaction. Figure 1 provides a schematic overview of the developmental stages of *C. purpurea* together with the key molecular processes associated with each stage.

## 2. Life Cycle and Pathogenic Process

The life cycle of *C. purpurea* begins with the germination of overwintered sclerotia, the fungal survival structures formed during the previous infection cycle [2]. Following a period of winter dormancy, sclerotia germinate in spring shortly before cereal flowering and produce stromata bearing perithecia, within which sexual ascospores are produced. These wind-dispersed ascospores constitute the primary inoculum and initiate infection upon reaching receptive stigmatic surfaces of flowering grasses, particularly rye and other economically important cereal crops [2,3,5].

After germination on the stigma, the infection hypha follows the pollen-tube pathway through the style and reaches the ovary within approximately 24 h, where biotrophic colonization is established [2]. During the subsequent sphacelial stage, characterized by extensive fungal proliferation within the developing ovary and the production of honeydew containing large numbers of asexual conidia, the developing ovary is progressively replaced by fungal tissue while remaining metabolically active. Approximately 7–9 days after infection, the fungus secretes a sugar-rich exudate known as honeydew, which contains large numbers of asexual conidia. These conidia serve as the secondary inoculum and are disseminated by insects attracted to the sugary exudate, rain splash and direct contact between neighbouring spikes, thereby promoting rapid disease spread during the flowering period [2,5,6].

As infection progresses, honeydew production gradually ceases and fungal growth shifts towards the differentiation of a mature sclerotium, which completely replaces the host ovary within 4–5 weeks after infection. This melanized, alkaloid-rich resting structure ensures fungal survival under adverse environmental conditions and constitutes the primary source of inoculum for the next growing season following germination and sexual reproduction (Figure 1) [7].

## 3. Initial Host Recognition, Adhesion, and Invasion

### 3.1. Spore Germination and Host Adhesion

Following deposition on receptive stigmatic surfaces, airborne ascospores initiate primary infection by adhering to stigmatic hairs and germinating under favourable environmental conditions, particularly high relative humidity and suitable temperatures. During the sphacelial stage, the production of asexual conidia enables secondary dissemination and contributes to disease propagation within flowering populations [2,5]. Infection by *C. purpurea* is highly specialized and predominantly restricted to floral organs, indicating that successful germination is likely influenced by host-derived chemical and structural cues present on the stigmatic surface. Although the molecular basis of this specificity remains largely unknown, current evidence suggests that host recognition involves signalling events that allow the fungus to establish compatibility with the host and subsequently follow the pollen-tube pathway [2].

Adhesion to the host surface constitutes a critical step in infection establishment. Whereas many phytopathogenic fungi rely on hydrophobins and adhesins to attach to plant surfaces, *C. purpurea* appears to employ a different strategy. Functional characterization of the class II hydrophobin CPPH1 demonstrated that deletion of the corresponding gene does not significantly affect conidial hydrophobicity or virulence, and conidia lack the rodlet layer typically associated with class I hydrophobins, suggesting that conventional hydrophobin-mediated adhesion mechanisms are not essential for host colonization [8,9,10]. These findings suggest that successful attachment depends predominantly on specific recognition of stigmatic tissues rather than on hydrophobic interactions.

After attachment, spores produce a short germ tube that penetrates directly through the thin cuticle of the stigmatic epidermis. Unlike many fungal pathogens, *C. purpurea* does not differentiate specialized infection structures such as appressoria, and host penetration is believed to rely primarily on the localized secretion of cell wall-degrading enzymes rather than on mechanical force [2,5]. The infection hypha subsequently grows intracellularly through the stigmatic hairs and advances in a highly polarized manner along the transmitting tissue of the style, following the route normally occupied by the pollen tube until it reaches the ovary, where biotrophic colonization is initiated [5,11].

### 3.2. Ovary Penetration and Colonization

The transition from stigma penetration to ovary colonization represents a critical step in the infection process of *C. purpurea*, as the fungus must reach and invade the reproductive tissues while maintaining a compatible interaction with the host. Hyphal growth occurs through the stigmatic hairs and transmitting tissue toward the developing ovary as compact bundles with limited branching. This progression closely resembles the pathway followed by the pollen tube, allowing the fungus to reach the basal region of the ovary [5,12]. Unlike many phytopathogenic fungi, *C. purpurea* does not form specialized penetration structures such as appressoria, and host invasion is thought to involve localized enzymatic modification of host tissues rather than mechanical pressure [13]. The ability of the fungus to exploit the pollen tube pathway has been proposed as a key adaptation underlying its strict specificity for floral tissues [14].

During colonization of the stigma and ovary, *C. purpurea* develops both intercellularly and intracellularly within host tissues, with hyphal growth associated with localized modification of the middle lamella and host cell walls. Unlike many obligate biotrophic fungi, *C. purpurea* does not appear to develop specialized feeding structures such as haustoria, but instead establishes a close association with living host cells during fungal expansion [2,7]. Once the ovary is reached, fungal growth becomes progressively more extensive, with increased branching and colonization of reproductive tissues, ultimately leading to the development of the sphacelial stage [6].

Successful ovarian colonization is associated with extensive remodelling of host cell wall components and the expression of multiple cell wall-degrading enzymes (CWDEs). Rather than causing extensive tissue maceration, these enzymes promote selective remodelling of plant cell wall structures, facilitating fungal progression while preserving host tissue viability, a characteristic consistent with the biotrophic lifestyle of the pathogen [2,11,15]. Among the most relevant CWDEs are cellulases, which hydrolyse the β-1,4-glucosidic linkages of cellulose, the principal structural component of the plant cell wall. The *C. purpurea* genome encodes multiple cellulases belonging to different glycosyl hydrolase families, whose expression is temporally regulated during infection. Dual RNA-seq analyses identified the cellobiohydrolase Cel1 (also designated Cbh1) as highly induced during the initial stages of infection. This expression pattern is consistent with a potential role in the controlled modification of host cellulose; however, direct functional evidence linking Cel1 activity to host tissue colonization is currently lacking [16].

Xylanases are also associated with host colonization. The xylanolytic system of *C. purpurea*, including the genes *cpxyl1* and *cpxyl2*, is actively expressed during infection of rye floral tissues, with cytological evidence demonstrating xylanase secretion in infected areas [2]. Transcriptomic analyses revealed significant induction of carbohydrate-active enzymes (CAZymes), including GH10 and GH11 xylanases as well as GH5 and GH7 cellulases, during stages of ovarian colonization [11,17,18]. Although these findings support a role for xylan degradation during infection, the causal contribution of individual xylanases to virulence remains to be established.

Pectin modification also plays an important role during tissue invasion. The *cppg1* and *cppg2* genes encode endopolygalacturonases that hydrolyse pectic components of the middle lamella, facilitating intercellular penetration and early tissue colonization. Functional studies have shown that disruption of these polygalacturonase genes reduces virulence, supporting a contribution of these enzymes to successful infection [15,19]. Together, the coordinated activity of cellulases, xylanases and polygalacturonases is consistent with a role for cell-wall remodelling in ovarian colonization, although the causal contribution of individual enzymes is supported to different extents by the available evidence.

Ovarian colonization marks the onset of the sphacelial stage, characterized by extensive proliferation of fungal biomass within the infected ovary [2,5].

### 3.3. Host Immune Responses and Fungal Modulation

Following successful colonization of the ovary, *C. purpurea* must establish a compatible interaction with the host while avoiding immune responses that would compromise fungal growth. Plant immunity relies on a two-tiered defence system. The first layer is initiated through the recognition of pathogen-associated molecular patterns (PAMPs) by pattern recognition receptors (PRRs), triggering PAMP-triggered immunity (PTI). In susceptible interactions, pathogen-mediated suppression or circumvention of PTI can contribute to a state of effector-triggered susceptibility (ETS), thereby favouring pathogen colonization. Conversely, recognition of these effectors by resistance (R) proteins activates effector-triggered immunity (ETI), frequently associated with a hypersensitive response (HR) involving localized cell death, which is particularly detrimental to biotrophic pathogens that depend on living host cells for nutrition [20].

For decades, *C. purpurea* was assumed to colonize cereal ovaries without being detected by the host, an idea supported by the absence of visible defence symptoms and by the pollen tube mimicry hypothesis, which proposes that the fungus may exploit processes associated with pollen tube growth and pollen–stigma interactions to facilitate compatibility with the host [2,7,19]. However, the first dual-transcriptome study of the *C. purpurea*–*Secale cereale* interaction demonstrated that the host actively responds to infection. These findings challenged the long-standing view that *C. purpurea* is largely undetected by the host during the early stages of infection. Defence-related genes, including a β-1,3-glucanase, a peroxidase and homologues of the disease resistance protein Rg4, were significantly upregulated in ovaries infected with the wild-type strain.

The virulence-attenuated mutants *Δcptf1* and *Δcpcdc42* elicited partially distinct transcriptional responses compared with the wild-type strain. Both mutants were associated with increased expression of a chitinase, whereas *Δcptf1* showed increased expression of genes involved in the biosynthesis of antifungal hydroxamic acids together with a xylanase inhibitor, while *Δcpcdc42* was associated with increased expression of a glutathione S-transferase and a reticuline oxidase-like protein. These differential transcriptional responses are consistent with a role for the corresponding fungal gene products in modulating host defence, although the precise molecular mechanisms involved remain to be elucidated.

The *C. purpurea* secretome contains numerous small secreted proteins (SSPs), several of which have been proposed as candidate effectors based on their predicted properties and expression during infection.

Genome-wide bioinformatic analyses identified 470 putative effector candidates, highlighting the remarkable expansion of the secreted effector repertoire in *C. purpurea*. These candidates should be distinguished from experimentally validated effectors, as their identification is primarily based on sequence and secretion-related predictions. Transcriptomic analyses further showed that 25 of the 150 most highly expressed fungal genes in planta (17%) encode putative SSPs, and that several of these genes are downregulated in the *Δcptf1* mutant, providing expression-based evidence for their association with infection but not, by itself, establishing a causal role in virulence [11].

Functional characterization provides stronger evidence for a role in virulence for a subset of these candidates. For example, deletion of *cp1105* reduced the infection rate to 68% of that observed for the wild-type strain, indicating that Cp1105 contributes to successful infection, although its molecular function and whether it directly modulates host immunity remain unknown. Similarly, deletion of *cp8623* reduced the infection rate to 70% of that of the wild type, indicating that Cp8623 also contributes to infection. Cp8623 contains a predicted LysM domain with similarity to those found in the well-characterized fungal effectors Ecp6 from *Cladosporium fulvum* and Slp1 from *Magnaporthe oryzae*. This sequence-based similarity suggests that Cp8623 may participate in chitin sequestration and potentially interfere with chitin-triggered host immunity; however, this proposed activity has not been directly demonstrated. Thus, although the reduced infection phenotype supports a role for Cp8623 in virulence, it does not establish the specific molecular mechanism underlying this phenotype.

In addition to the expression of secreted proteins, *C. purpurea* infection is associated with alterations in host hormonal signalling, which may represent an additional component of the host–pathogen interaction. Transcriptomic studies have shown alterations in auxin-responsive genes in infected ovaries, while genetic evidence demonstrates that fungal-derived cytokinins are required for full virulence, indicating that hormonal regulation contributes to the establishment of a compatible interaction [11].

Collectively, current transcriptomic and genetic evidence supports a model in which the host perceives *C. purpurea* but mounts a defence response that is insufficient to restrict fungal colonization. Evidence suggests that successful establishment and maintenance of biotrophy are likely to involve multiple secreted proteins and alterations in host physiology. However, the extent to which these components act coordinately or redundantly, and the specific mechanisms by which they influence host–pathogen compatibility, remain unresolved. Together, these findings support a model in which secreted proteins and alterations in host physiology may contribute to the maintenance of a compatible interaction with living host tissues throughout floral colonization [2,11,20].

The main fungal genes and proteins currently associated with host colonization, virulence, development, and biosynthetic processes are summarized in Table 1.

## 4. Biotrophic Colonization and Honeydew Production

### 4.1. Establishment of Biotrophy

The establishment of biotrophy represents a pivotal stage in the life cycle of *C. purpurea*, allowing the fungus to colonize host tissues while preserving host cellular activity during the biotrophic phase [11]. Unlike necrotrophic pathogens that exploit host cell death for nutrient acquisition, *C. purpurea* depends on the prolonged viability of ovarian tissues throughout much of its infection cycle, requiring the maintenance of a compatible interaction with the host [14].

During the sphacelial stage, fungal hyphae progressively replace the reproductive tissues of the ovary with a compact mycelial mass associated with honeydew production, a sugar-rich exudate containing large numbers of secondary conidia that promotes pathogen dissemination. Honeydew production ceases approximately two weeks after infection, coinciding with the onset of sclerotium differentiation [2]. Histological studies have shown that hyphae grow predominantly through the intercellular spaces of ovarian tissues and establish an intimate association with living host cells without producing differentiated feeding structures such as haustoria, as observed in rusts and powdery mildews [2,7]. This distinctive mode of colonization suggests that *C. purpurea* relies on alternative nutrient acquisition strategies that differ from classical haustorial biotrophs.

The maintenance of biotrophy involves coordinated fungal growth together with continuous nutrient acquisition while preserving host tissue integrity. Transcriptomic studies have shown that genes involved in nutrient transport, carbohydrate acquisition, and fungal metabolism are highly expressed during this stage, consistent with the metabolic demands of sustained fungal proliferation within the living ovary and preparing the developmental transition towards honeydew production and subsequent sclerotial differentiation [11,14].

### 4.2. Honeydew Composition and Function

A hallmark of the sphacelial stage is the abundant production of honeydew, a viscous sugar-rich exudate that emerges from infected florets and contains large numbers of secondary conidia. Honeydew production coincides with rapid fungal proliferation within the ovary and represents a major mechanism for secondary dissemination of *C. purpurea* during the flowering period [2,26].

Honeydew consists mainly of soluble carbohydrates, including glucose, fructose and sucrose, together with amino acids and other nitrogen-containing compounds, reflecting the extensive metabolic activity of the fungus during the sphacelial stage. This nutrient-rich composition attracts numerous floral visitors, particularly dipteran and coleopteran insects, which transport conidia to susceptible flowers and contribute to disease spread [27].

The conidia suspended in honeydew constitute the major source of secondary inoculum. These unicellular, hyaline spores readily germinate on receptive stigmas, facilitating repeated transmission events during the same flowering season. Insect-mediated dispersal complements passive dissemination through rain splash and direct contact between infected and healthy floral structures [2,24,26].

Despite its central role in disease epidemiology, the molecular composition of honeydew remains only partially characterized. Although several secreted proteins are expressed during the sphacelial stage, their presence and specific functions within honeydew have not yet been fully established [11]. Future proteomic and metabolomic analyses of honeydew may provide new insights into fungal secretion strategies, nutrient acquisition, and interactions with insect-mediated dispersal.

### 4.3. Alterations in Host Hormonal Signalling

Hormonal regulation constitutes an important component of the compatible interaction established between *C. purpurea* and its host. During the sphacelial stage, fungal proliferation within the ovary is accompanied by profound alterations in host physiology, including changes in phytohormone homeostasis [11]. Because phytohormones coordinate plant development, nutrient allocation and defence responses, these hormonal changes may contribute to the physiological conditions associated with biotrophic colonization [25].

Among the phytohormones affected during infection, cytokinins (CKs) play a prominent role in the interaction between *C. purpurea* and its host. Studies have shown that CK levels increase significantly in infected tissues, with this accumulation mainly resulting from the ability of the fungus to synthesize its own cytokinins through two distinct pathways: a de novo biosynthetic pathway involving the bifunctional enzyme CpIPT-LOG and a second pathway associated with the modification and degradation of transfer RNA (tRNA) catalysed by the tRNA-isopentenyltransferase CptRNA-IPT [23,28,29]. The latter pathway accounts for virtually all cis-zeatin-type CKs produced by the fungus [29]. Increased cytokinin production may contribute to maintaining host cell viability required for biotrophic growth and to establishing a metabolic sink that redirects nutrients towards the infection site [23,28].

However, these proposed functions remain to be experimentally established, as increased CK levels alone do not demonstrate the underlying mechanisms. Stronger functional evidence comes from genetic analyses showing that a double deletion mutant (ΔΔipt-log/tRNA-ipt) unable to synthesize CKs was nearly apathogenic, with hyphae detected in only 21% of inoculated ovaries and no visible disease symptoms [23]. These findings demonstrate that fungal CK biosynthesis contributes to full virulence; however, the mechanisms through which fungal CKs promote biotrophic colonization remain unresolved [20,23].

Auxins, particularly indole-3-acetic acid (IAA), also appear to participate in the interaction. Increased IAA levels have been detected following inoculation with *C. purpurea* [18]. In addition, several studies have suggested that the fungus is able to synthesise IAA from tryptophan through pathways similar to those described in other auxin-producing fungi. The importance of tryptophan metabolism for pathogenicity is supported by mutagenesis studies showing that mutations in the *TrpE* gene are non-pathogenic; however, this phenotype does not by itself establish a specific role for fungal IAA biosynthesis [2]. Altered auxin levels may contribute to changes in infected ovary development and potentially influence fungal colonization, although the mechanisms underlying these effects remain unclear [2,18].

In addition to growth-promoting hormones, defence-associated hormonal pathways are also affected during infection. The balance between salicylic acid (SA)- and jasmonic acid (JA)/ethylene-mediated signalling is a major determinant of the outcome of plant–pathogen interactions [28]. In general, SA signalling is associated with defence against biotrophic pathogens, whereas JA- and ethylene-mediated signalling is more effective against necrotrophic pathogens [29]. Evidence suggests that *C. purpurea* interferes with these signalling pathways. For example, studies in wheat have shown that JA levels decrease following fungal inoculation, whereas SA levels do not exhibit informative changes, suggesting active modulation of these defence pathways. However, the molecular mechanisms by which *C. purpurea* modulates or circumvents these responses remain to be elucidated [18]. These observations provide evidence of altered hormone signalling during infection, but do not by themselves demonstrate active fungal suppression of JA/SA pathways. The molecular mechanisms by which *C. purpurea* may modulate or circumvent these responses therefore remain to be elucidated.

Collectively, these findings indicate that alterations in hormone homeostasis are associated with sustained biotrophic growth and disease development, while the extent and mechanisms of fungal control over these hormonal changes remain unresolved [18,30].

## 5. Transition from Biotrophy to Sclerotial Differentiation

### 5.1. Developmental Reprogramming and Morphological Differentiation

The transition from the sphacelial phase to sclerotium formation represents a major developmental switch in the life cycle of *C. purpurea*, involving extensive transcriptional, metabolic, and morphological reprogramming. This transition generally occurs around 10–14 days after infection, when secondary conidium production declines and fungal development shifts from active proliferation towards the differentiation of a compact resting structure [31,32].

During early sclerotial development, vegetative hyphae undergo extensive aggregation and differentiation, forming a dense pseudoparenchymatous tissue that progressively replaces the sphacelial mycelial organization. This process is accompanied by a redistribution of cellular resources from growth-associated metabolism towards storage and protection mechanisms required for long-term survival. Lipidomic analyses have demonstrated that mature sclerotia accumulate large amounts of neutral lipids, particularly triglycerides enriched in unsaturated fatty acids such as linoleic acid, which provide an essential energy reserve during dormancy and subsequent germination [7,32,33]. These metabolic reserves highlight the profound shift from nutrient acquisition during biotrophy towards long-term survival and developmental competence.

A characteristic feature of sclerotial maturation is the progressive pigmentation of the external layers, resulting in the typical dark purple-black coloration of mature *C. purpurea* sclerotia. In many filamentous fungi, pigmentation and environmental protection are associated with melanin biosynthesis; however, the characteristic coloration of *C. purpurea* sclerotia is mainly associated with the accumulation of ergochrome pigments and other phenolic compounds. These pigments are produced through specific secondary metabolic pathways that are activated during the late stages of sclerotial development. Their accumulation has been proposed to contribute to protection against environmental stresses, including ultraviolet radiation, desiccation and microbial antagonism, although their precise ecological functions remain incompletely understood [34].

Together, these morphological and metabolic changes reflect the profound developmental reprogramming associated with sclerotial differentiation, enabling *C. purpurea* to transition from active growth during infection towards a dormant survival structure.

### 5.2. Molecular Regulation of Sclerotium Formation

Sclerotial differentiation involves developmental signalling pathways that regulate hyphal aggregation, oxidative signalling and metabolic adaptation. Although the complete regulatory network controlling this transition remains poorly characterized in *C. purpurea*, studies in this species and related filamentous fungi have implicated reactive oxygen species (ROS)-mediated signalling in fungal differentiation and development [11].

In *C. purpurea*, NADPH oxidase complexes participate in both host interaction and developmental differentiation. The catalytic subunits CpNox1 and CpNox2, together with the tetraspanin CpPls1, regulate localized ROS production required for developmental processes. Deletion of *cpnox1* impairs sclerotium formation, demonstrating that CpNox1 contributes to normal sclerotial differentiation. Conversely, mutants affected in cpnox2 or cppls1 retain the ability to produce sclerotia, suggesting functional specialization among NADPH oxidase complexes during fungal development and host interaction [21,22]. These findings suggest that ROS signalling is not simply a consequence of fungal development but acts as a regulated developmental signal required for specific differentiation processes [22].

The developmental transition is also strongly influenced by nutritional signals. Early physiological studies demonstrated that nitrogen availability regulates the balance between sphacelial growth and sclerotial differentiation. Acidic amino acids, such as glutamate and aspartate, favour sclerotial development and ergot alkaloid accumulation, whereas their corresponding amides promote vegetative sphacelial growth [31]. These observations indicate that nutrient sensing acts as a critical checkpoint controlling the transition from active host colonization towards survival structure formation. Moreover, the close association between nutrient availability and sclerotial differentiation suggests that developmental decisions in *C. purpurea* are tightly coordinated with the activation of specialized secondary metabolism.

### 5.3. Metabolic Specialization and Ergot Alkaloid Biosynthesis

Sclerotium maturation is accompanied by a major metabolic specialization characterized by the activation of secondary metabolite pathways, particularly the biosynthesis of ergot alkaloids. These indole-derived compounds represent one of the most distinctive biochemical features of *C. purpurea* and accumulate predominantly during late stages of sclerotial development [1].

Ergot alkaloid biosynthesis is controlled by the Ergot Alkaloid Synthesis (EAS) gene cluster, a chromosomal region of approximately 68.5 kb comprising approximately 14 co-expressed genes that are activated under conditions favouring alkaloid production [1]. The first pathway-specific step is catalysed by dimethylallyltryptophan synthase (DMATS), encoded by dmaW, which catalyses the prenylation of L-tryptophan to generate 4-dimethylallyl-L-tryptophan (DMAT), thereby linking primary tryptophan metabolism to ergot alkaloid biosynthesis [1,5].

Subsequent enzymatic reactions involving methylation, oxidation, cyclization and reduction convert DMAT into chanoclavine-I, the first cyclic intermediate of the pathway. Further reactions generate agroclavine, elymoclavine and finally D-lysergic acid, the common precursor of lysergic acid amides and ergopeptines. Formation of the characteristic ergoline ring is catalysed by the cytochrome P450 monooxygenase CloA, encoded by cloA, which plays an essential role in the biosynthetic pathway [35,36].

Expression of the ergot alkaloid synthesis (EAS) cluster is associated with sclerotial maturation and appears to be influenced by developmental, nutritional, and epigenetic factors. Promoter analyses have identified binding sites for global regulators of fungal carbon, nitrogen, and phosphate metabolism, including CreA, AreA, PacC, and Nuc-1, indicating that environmental conditions contribute to the transcriptional regulation of alkaloid biosynthesis. In addition, inhibition of histone deacetylases significantly enhances ergot alkaloid production, indicating that chromatin-associated regulation contributes to control of EAS cluster activity [37].

The final steps involve condensation of D-lysergic acid with amino acids through non-ribosomal peptide synthetases (NRPSs). In *C. purpurea*, synthesis of the major ergopeptines requires the coordinated activity of LpsA, which determines the amino acid composition of the tripeptide moiety, and LpsB, which activates and incorporates D-lysergic acid into the biosynthetic complex. Variation in the amino acid composition of the tripeptide accounts for the structural diversity of ergopeptines produced by the fungus [38,39].

## 6. Sclerotial Germination and Sexual Reproduction

Following winter dormancy, sclerotia present near the soil surface germinate under favourable temperature and moisture conditions, producing one or more stromata that initiate the sexual phase of the life cycle [40,41]. Each stroma consists of a stalk (stipe) supporting a globose capitulum containing numerous perithecia. Within each perithecium, asci containing eight filamentous ascospores are formed, providing the primary inoculum responsible for initiating new infections in susceptible florets [2].

Stromatal development and ascospore release are tightly synchronized with the flowering of susceptible grasses, ensuring that ascospores are dispersed when receptive stigmas are available. This developmental and temporal coordination represents a key evolutionary adaptation that maximizes infection efficiency while maintaining the strict floral specificity that characterizes *C. purpurea* [2]. Germination occurs predominantly in sclerotia located near the soil surface, whereas deeply buried sclerotia exhibit a greatly reduced capacity to produce viable stromata, thereby limiting their contribution to the primary inoculum [40,41].

Thus, sclerotial germination links long-term survival with sexual reproduction and completes the disease cycle of *C. purpurea*. The coordination between dormancy, environmental conditions, stromatal development, and host flowering ensures pathogen persistence between growing seasons and contributes to its epidemiological success [2,39].

## 7. Conclusions and Future Perspectives

Over the past two decades, substantial advances in genomics, transcriptomics, functional genetics and metabolomics have considerably expanded our understanding of the biology of *C. purpurea*. Rather than representing a simple floral pathogen, *C. purpurea* has emerged as a highly specialized biotrophic fungus that coordinates host recognition, immune modulation, developmental reprogramming, and secondary metabolism to complete its complex infection cycle. The integration of multi-omics approaches has identified key molecular processes underlying floral colonization, including the contribution of secreted effectors, carbohydrate-active enzymes, hormonal manipulation, and regulatory networks controlling fungal differentiation and ergot alkaloid biosynthesis.

Despite these advances, several fundamental questions remain unresolved. The molecular mechanisms governing the earliest stages of host recognition and floral specificity are still poorly understood, and the host receptors and fungal ligands responsible for establishing compatibility have yet to be identified. Likewise, although numerous candidate effectors have been described, the biological functions of most secreted proteins remain unknown, and their potential functional redundancy complicates their characterization. Similarly, the molecular basis by which *C. purpurea* modulates host immune responses and hormonal signalling to maintain long-term biotrophy while avoiding defence responses that would compromise fungal growth remains poorly understood.

Important knowledge gaps also persist regarding fungal developmental transitions. The signalling pathways coordinating the shift from sphacelial growth to sclerotial differentiation, the integration of environmental and nutritional cues with fungal developmental programmes, and the regulatory networks linking sclerotial maturation with activation of the EAS biosynthetic cluster require further investigation. Likewise, although significant progress has been made in understanding ergot alkaloid biosynthesis, the mechanisms coordinating secondary metabolism with fungal development, chromatin dynamics, and host-derived signals remain incompletely characterized.

Nevertheless, the current understanding of *C. purpurea* biology remains limited by the relatively small number of comprehensive studies. Furthermore, differences in experimental designs, biological replication, sequencing strategies, bioinformatic workflows, and statistical methodologies may hinder direct comparisons among datasets. Expanding comparative studies involving diverse fungal isolates and host genotypes, together with robust functional validation, will be essential to strengthen current models of host colonization, fungal development, and secondary metabolism.

Future research should therefore complement descriptive transcriptomic approaches with functional analyses to dissect the regulatory networks governing host–pathogen interactions. The integration of comparative genomics, dual RNA-seq, spatial transcriptomics, proteomics, metabolomics, and targeted genome editing will provide a more comprehensive understanding of the molecular dialogue between *C. purpurea* and its host. Combining these approaches will be essential to resolve the functions of candidate effectors, define the regulatory circuits controlling developmental transitions, and clarify how environmental signals are translated into developmental and metabolic responses.

A deeper understanding of these processes will not only improve our knowledge of one of the most specialized biotrophic interactions among phytopathogenic fungi but will also have practical implications for agriculture and biotechnology. Identifying the molecular determinants of floral infection may facilitate the development of novel strategies for ergot disease management, while elucidating the regulation of ergot alkaloid biosynthesis could contribute to improving the controlled production of pharmaceutically valuable compounds. Consequently, *C. purpurea* represents a unique model system for understanding how fungal pathogens integrate host specialization, developmental plasticity, and secondary metabolism to establish successful biotrophic interactions.

## Figures and Tables

**Table 1:** Fungal genes and proteins associated with virulence, host colonization, development, and biosynthetic pathways in *Claviceps purpurea*.

Gene/Protein	Function/Mechanism	Evidence	Reference
Cel1/Cbh1	Cellulose degradation during early infection	Strongly induced during infection; function not demonstrated	[16]
*cpxyl1/cpxyl2*	Xylan degradation	Expression and secretion associated with infected floral tissues	[2]
*cppg1/cppg2*	Pectin degradation	Gene disruption reduces virulence	[15,19]
*cp1105*	Putative secreted effector	Deletion reduces infection to ~68% of WT	[11]
*cp8623*	LysM-containing putative effector	Deletion reduces infection to ~70% of WT	[11]
CpNox1	NADPH oxidase/ROS signalling	Deletion impairs sclerotium formation	[21,22]
CpNox2	NADPH oxidase/ROS signalling	Mutant retains sclerotium formation	[21,22]
CpPls1	Tetraspanin associated with ROS regulation	Mutant retains sclerotium formation	[21,22]
CplPT-LOG/CptRNA-IPT	Cytokinin biosynthesis	Double mutant is nearly apathogenic	[23,24,25]
*TrpE*	Tryptophan biosynthesis	Mutants are non-pathogenic	[2]

## Data Availability

No new data were created or analyzed in this study. Data sharing is not applicable to this article.

## Abbreviations

The following abbreviations are used in this manuscript:

SSPs	Small Secreted Proteins
CWDEs	Cell Wall-Degrading Enzymes
CAZymes	Carbohydrate-Active Enzymes
GH	Glycosyl Hydrolase
Cel1/Cbh1	Cellobiohydrolase 1
PAMPs	Pathogen-Associated Molecular Patterns
PRRs	Pattern Recognition Receptors
PTI	PAMP-Triggered Immunity
ETS	Effector-Triggered Susceptibility
ETI	Effector-Triggered Immunity
R proteins	Resistance Proteins
HR	Hypersensitive Response
LysM	Lysin Motif
CKs	Cytokinins
CpIPT-LOG	Bifunctional Isopentenyltransferase–LONELY GUY enzyme
tRNA	Transfer RNA
CptRNA-IPT	tRNA Isopentenyltransferase
IAA	Indole-3-Acetic Acid
GA	Gibberellic Acid
SA	Salicylic Acid
JA	Jasmonic Acid
ROS	Reactive Oxygen Species
NADPH	Nicotinamide Adenine Dinucleotide Phosphate
EAS	Ergot Alkaloid Synthesis
kb	Kilobase
DMATS	Dimethylallyltryptophan Synthase
dmaW	Dimethylallyltryptophan Synthase gene
DMAT	4-Dimethylallyl-L-Tryptophan
NRPSs	Non-Ribosomal Peptide Synthetases
LpsA	Lysergyl Peptide Synthetase A
LpsB	Lysergyl Peptide Synthetase B
RNA-seq	RNA Sequencing
Dual RNA-seq	Dual RNA Sequencing
Multi-omics	Multi-omics Approaches
